# Network meta-analysis of randomised trials of pharmacological, psychotherapeutic, exercise and collaborative care interventions for depressive symptoms in patients with coronary artery disease: hybrid systematic review of systematic reviews protocol

**DOI:** 10.1186/s13643-019-0985-9

**Published:** 2019-03-16

**Authors:** Frank Doyle, Kenneth Freedland, Robert Carney, Peter de Jonge, Chris Dickens, Susanne Pedersen, Jan Sorensen, Martin Dempster

**Affiliations:** 1 Department of Health Psychology, Royal College of Surgeons in Ireland, 123 St Stephen’s Green, Dublin 2, Ireland; 2 School of Psychology, Queen’s University Belfast, University Road, Belfast, BT71NN Northern Ireland, UK; 3 Washington University School of Medicine, St. Louis, USA; 4 University of Groningen, Groningen, Netherlands; 5 University of Exeter, Exeter, UK; 6 University of Southern Denmark, Odense, Denmark; 7 Royal College of Surgeons in Ireland, Dublin, Ireland; 8 Queen’s University Belfast, Belfast, UK

**Keywords:** Depression, Coronary artery disease, Network meta-analysis, Systematic review, Randomised controlled trial, Intervention

## Abstract

**Background:**

Depression is common in patients with coronary artery disease (CAD) and is associated with poorer outcomes and higher costs. Several randomised controlled trials (RCTs) targeting depression, of various modalities (including pharmacological, psychotherapeutic and other approaches), have been conducted and summarised in pairwise meta-analytic reviews. However, no study has considered the cumulative evidence within a network, which can provide valuable indirect comparisons and information about the relative efficacy of interventions. Therefore, we will adopt a review of review methodology to develop a network meta-analysis (NMA) of depression interventions for depression in CAD.

**Methods:**

We will search relevant databases from inception for systematic reviews of RCTs of depression treatments for people with CAD, supplementing this with comprehensive searches for recent or ongoing studies. We will extract data from and summarise characteristics of individual RCTs, including participants, study characteristics, outcome measures and adverse events. Cochrane risk of bias ratings will also be extracted or if not present will be conducted by the authors.

RCTs that compare depression treatments (grouped as pharmacological, psychotherapeutic, combined pharmacological/psychotherapeutic, exercise, collaborative care) to placebo, usual care, waitlist control or attention controls, or directly in head-to-head comparisons, will be included. Primary outcomes will be the change in depressive symptoms (summarised with a standardised mean difference) and treatment acceptability (treatment discontinuation: % of people who withdrew). Secondary outcomes will include change in 6-month depression outcomes, health-related quality of life (HRQoL), mortality, cardiovascular morbidity, health services use and adverse events. Secondary analyses will form further networks with individual anti-depressants and psychotherapies.

We will use frequentist, random effects multivariate network meta-analysis to synthesise the evidence for depression intervention and to achieve a ranking of treatments, using Stata. Rankograms and surface under the cumulative ranking curves will be used for treatment ranking. Local and global methods will evaluate consistency. GRADE will be used to assess evidence quality for primary outcomes.

**Discussion:**

The present review will address uncertainties about the evidence in terms of depression management in CAD and may allow for a ranking of treatments, including providing important information for future research efforts.

**Systematic review registration:**

PROSPERO CRD42018108293

**Electronic supplementary material:**

The online version of this article (10.1186/s13643-019-0985-9) contains supplementary material, which is available to authorized users.

## Background

Depression is common in patients with coronary artery disease (CAD) and negatively impacts on clinically important outcomes. For example, the prevalence of major depression post-myocardial infarction reported to be 12–20%, with up to 38% reporting elevated depressive symptoms [1, 2], which is higher than seen in community samples [3]. Both major depression and depressive symptoms have been associated with increased mortality, morbidity, poorer quality of life, higher health service utilisation and increased healthcare costs [1, 4, 5]. Depression in CAD is therefore an important treatment target, and treatment interventions should be thoroughly evaluated.

While a substantial body of research exists in terms of depression interventions in those with CAD [4, 6–11], the effects of both pharmacological and psychological interventions are typically small [6, 8, 12]. Indeed, the effect sizes from these studies may even be smaller than those seen in general population samples [13] or other chronic conditions such as diabetes [14]. While exercise interventions for depression in general populations are effective [15] and show significant promise for depression in CAD [16, 17], exercise is often provided as part of multi-component cardiac rehabilitation programmes [10], which also contain some form of psychological intervention and risk factor education. Yet participants enrolled in such programmes are rarely enrolled primarily for depression treatment [10]. Furthermore, depression is a known impediment to joining, and dropping out, of such programmes [18, 19], meaning that comparison among such studies is unlikely to be appropriate or informative. It is therefore difficult to determine the efficacy of exercise alone. Furthermore, the contents of psychological interventions—which often are described as having a stress management component—are not always clear and can be highly variable [6, 9, 10, 12]. It is therefore difficult to compare the effects of these interventions and make recommendations regarding the best treatment choices for depression in the CAD population.

While ideally, direct comparisons of depression treatments for CAD would provide clinicians and policy-makers with excellent evidence for efficacy and acceptability of depression interventions, currently, this data is not available. However, there may be sufficient evidence of such interventions to provide indirect comparisons [20]. Network meta-analysis (NMA) is an advanced technique in that it allows both direct comparisons (as does pairwise meta-analysis), but also indirect comparisons for treatments that have not been made in a head-to-head format, and is recommended when competing interventions are used [20–25]. It can therefore provide crucial evidence for clinicians that can account for multiple outcomes simultaneously and even provide a ranking of treatments (e.g. treatment efficacy and acceptability to patients). Recent examples include (both direct and indirect) comparisons of behavioural and pharmacological treatments for smoking cessation [26] and comparison of second-generation anti-depressants [13], among others.

Given the plethora of reviews and pairwise meta-analyses in the field of CAD and depression (e.g. [4, 6–11, 25, 27], yet the significant uncertainty that still remains in terms of comparing treatments, it appears that NMA may provide an important opportunity to summarise the current depression intervention literature for those with CAD, in terms of both efficacy and acceptability (the latter outcome being largely neglected in pairwise meta-analyses). Indeed, it has been noted that in the absence of several direct head-to-head RCTs of competing interventions, or when using more than one outcome to rank treatments, NMA is the best available approach [20, 28]. A recent NMA of second-generation anti-depressants excluded trials of patients with serious concomitant medical illness [13], such as CAD, leaving a significant gap in the psychosomatic literature.

However, performing NMA of psychological interventions, where the content of interventions is unclear, is not necessarily methodologically sound (or indeed for pairwise meta-analysis)—we therefore will not address such interventions. However, established psychotherapies for depression, which follow a theoretical framework and standard procedures and are delivered by a trained psychotherapist (which are often manualised, allowing for replication), should be more comparable and are frequently meta-analysed and indeed included in NMA [29–31]. Furthermore, collaborative care, a multi-professional, structured approach to depression management and enhanced communication and follow-up [11], can encompass both combined psychotherapy and anti-depressant usage. Such treatments—anti-depressants, psychotherapies and collaborative care—have all been investigated in recent RCTs in patients with CAD, have been combined in other pairwise and network meta-analyses [11, 31, 32], and should therefore be comparable for NMA. While exercise is an established treatment for depression [15], as outlined above, it is usually encompassed in (heterogeneous) cardiac rehabilitation programmes, but where it studied alone it could be compared to other interventions.

## Objectives

The main objective of this NMA is to compare the best established treatment(s) for depression in patients with CAD in terms of efficacy (or effectiveness, in studies using non-placebo comparators) and acceptability, with several secondary outcomes also considered. Cardiac rehabilitation will not be included, as this is currently undergoing investigation elsewhere [33], but more importantly because cardiac rehabilitation per se would not be considered a front-line depression intervention. The PICO is as follows:

- Participants: patients with CAD and elevated depressive symptoms (clinical diagnosis of depression [any clinical diagnosis of depression that is made by a clinician or by structured diagnostic interview], or scoring above threshold on any validated depression scale) enrolled in randomised trials for depression treatment in any setting, excluding cardiac rehabilitation
- Interventions: any established treatment (groupings) for depression, including pharmacotherapy, psychotherapy, exercise and collaborative care
- Comparison: placebo groups, usual care or waitlist control, attention control groups
- Outcomes:
  - o Primary: change in depressive symptoms at 8-weeks;, acceptability (% of patients who discontinue treatment),
  - o Secondary: change in depressive symptoms at 26 weeks, depression response (≥ 50% change in total score on observer or self-report rating depression scale [13]), health-related quality of life (HRQoL), mortality (all-cause, cause specific), re-infarction and other cardiac complications, health services use, adverse events

## Methods and analysis

We use the PRISMA-P and PRISMA extension statement for NMA guidelines for reporting this protocol [28, 34, 35] (see Additional file 1). This protocol has been registered on PROSPERO (CRD42018108293).

### Criteria for considering studies for this review: eligibility criteria

#### Study types

We will include all randomised trials of interventions for depression in patients with CAD, including pharmacotherapy, psychotherapy, exercise or collaborative care, which use a validated depression scale or diagnostic interviews as an outcome measure, and report a (potential) change in depressive symptoms from baseline or pre-treatment to post-treatment. Cross-over or cluster RCTs will be included, but quasi-RCTs or those focusing on cardiac rehabilitation, or psychological interventions that are not established psychotherapies delivered by trained therapists, will be excluded. Studies will be published or summarised in peer-reviewed journals or review articles, or RCT registries, in the English language. If relevant RCTs are not summarised sufficiently in English in any found review, we will exclude them. Although this may lead to publication bias, exclusion of non-English articles is typical in this field [4, 11, 12, 27].

#### Participants

Participants will be aged 18 years and over, diagnosed with CAD (including acute coronary syndrome, angina, angiographically confirmed coronary disease, receipt of percutaneous coronary intervention or coronary bypass graft), and be enrolled in an RCT that targets elevated depression (clinical diagnosis of depression or scoring above threshold on a validated scale) as either a primary or secondary outcome, with a validated depression scale score at baseline or pre-enrolment, and post-intervention, from which to calculate change scores. If RCTs have combined CAD and other patients with coronary heart disease diagnoses, authors will be requested to provide estimates for CAD patients only. Otherwise, if ≥ 70% of participants have a diagnosis of CAD, the overall trial estimates will be included. We will exclude studies where ≥ 20% of people have bipolar or psychotic depression, or where all participants have concurrent secondary psychiatric diagnoses [36].

#### Intervention types

We will include the following groups of interventions:

- Pharmacotherapies: selective serotonin re-uptake inhibitors, monoamine oxidase inhibitors, tricyclic anti-depressants, anxiolytics etc.
- Psychotherapy trials delivered by trained therapists: cognitive-behavioural therapy, interpersonal psychotherapy, mindfulness, acceptance and commitment therapy, behavioural therapy, cognitive remediation, cognitive stimulation therapy, dialectical behaviour therapy, family systems therapy, integrative psychotherapy, multimodal therapy, positive psychology interventions, problem-solving therapy, psychodynamic psychotherapy, supportive therapy and counselling.
- Combination therapies (pharmacotherapies with psychotherapy): a combination of the above two interventions
- Exercise: specific exercise targeting depression, but not (multi-component) cardiac rehabilitation.
- Collaborative care: interventions that are labelled by trial authors specifically as collaborative care programmes and meet accepted criteria [11, 37, 38](depression care interventions including all of the following: a multi-professional approach, structured management plan, scheduled follow-up, enhanced inter-professional communication).

Given the previous evidence supporting these depression interventions in CAD, we assume that included patients are equally likely to have been randomised to any of the interventions described above [28]. As recommended, unspecified interventions (e.g. complementary and alternative therapies) may be included, post hoc, in the network, if they are deemed to supply vital information for increasing precision of the results [28], but otherwise will not be included. Such additions will be carefully documented and reported.

#### Comparison groups

**Fig. 1:**
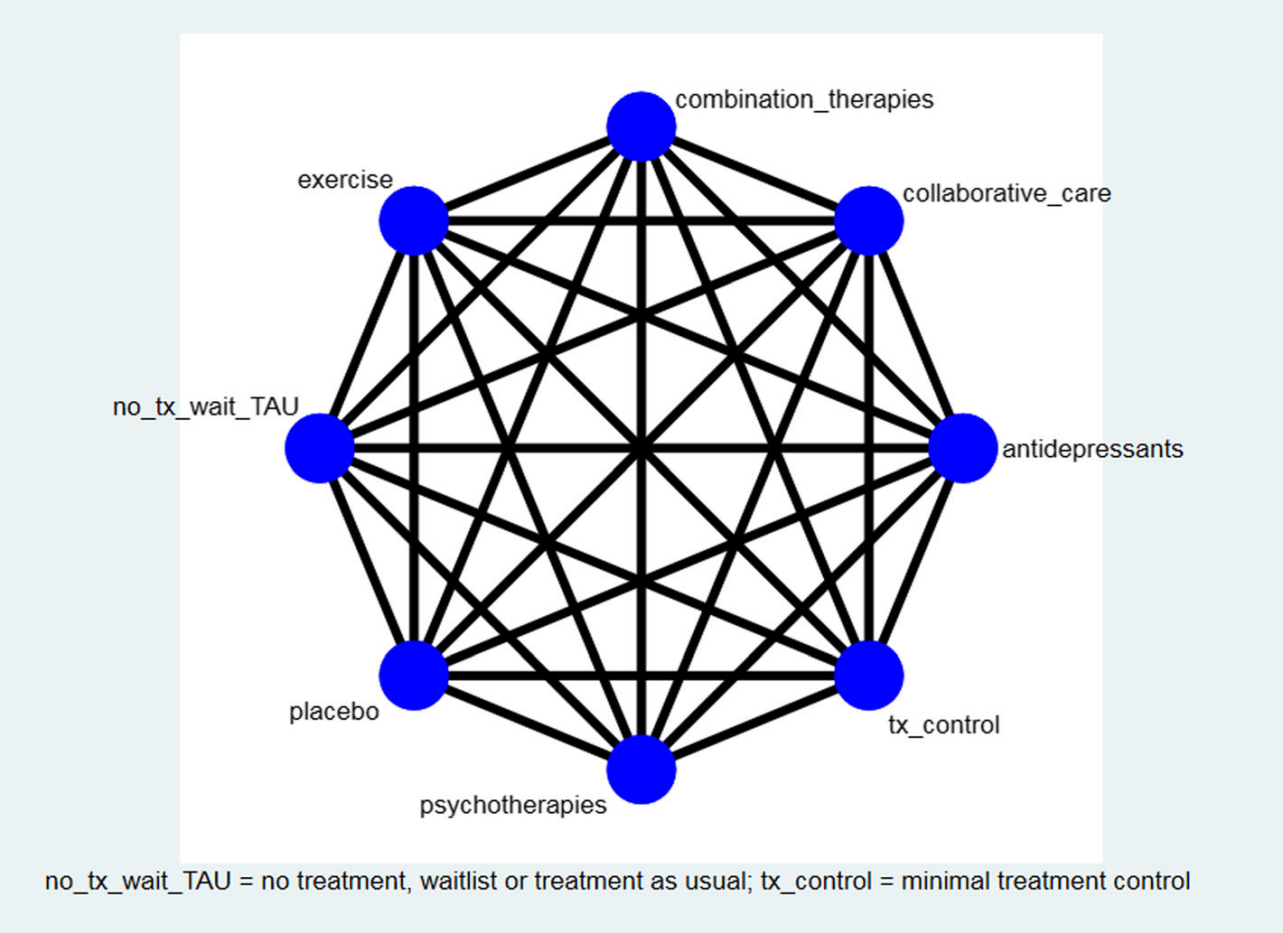
Network of possible comparisons between intervention and comparator groupings

RCTs which compare to pill placebo control groups, or other comparator groups [39], or directly in a head-to-head format, will be included. Non-placebo control groups can differ substantially from various forms of control designs in psychotherapeutic research, with considerable implications for study effect sizes and conducting of NMA [39, 40]. Previous research has shown that other comparator groups can include no treatment, treatment as usual, waitlist control (experimental treatment is offered after post-treatment assessment), minimal treatment control (fewer than four treatments), active comparator (evidence-based treatment), non-specific factor component control (equivalent time with interventionist but without specific therapeutic content), specific factor component control (equivalent time with interventionist with different or reduced therapeutic content), no treatment control, patient’s choice (personal preference for offered treatments) [39, 40]. However, there is likely to be far more limited literature in the current area of depression in CAD. Therefore, following a recommended framework [40], these comparator groups will be combined to allow for more appropriate comparison, as follows: (1) pill placebo; (2) no treatment, waitlist or treatment as usual; and (3) minimal treatment control, active comparator, specific and non-specific factors treatment control. Where the classification of comparison groups is unclear, authors will be contacted to request further detail. If this is not forthcoming, we will use another grouping of ‘unclear’ or eliminate the study from the NMA. The currently proposed network plot of all possible comparisons is shown in Fig. 1.

### Outcomes

#### Primary outcomes

We will adopt two primary outcomes, similar to those of a recent NMA of anti-depressant therapy and other reviews in similar areas [6, 11, 13], as follows:

1. Efficacy (effectiveness)-response (continuous): change in depressive symptoms (as measured by validated tools and summarised with standardised mean difference [SMD]) at week 8 post-intervention (or closest measure to 8 weeks that is available, between 4 and 16 weeks)
2. Acceptability: defined as the proportion of participants who discontinue treatment (for any reason [such reasons will be recorded where available]) between 4 and 16 weeks after initial intervention.

#### Secondary outcomes

1. Efficacy (effectiveness)-response (continuous): change in depressive symptoms (as measured by validated tools and summarised with standardised mean difference (SMD)) at week 26 post-intervention (or closest measure to 26 weeks that is available, between 20 and 30 weeks)
2. HRQoL: change in HRQoL scores summarised using SMD. If HRQoL scores are not available, then generic QoL scores will be used as appropriate.
3. Mortality: the proportion of participants who die during or after treatment, for the longest duration of available data. All-cause mortality will be preferred, but substituted with cardiovascular mortality if unavailable. If sufficient data is available, both all-cause and cause-specific mortality will be modelled separately.
4. Morbidity: the proportion of participants who have re-infarction or recurrent PCI or CABG, or other acute coronary events/interventions, during or after treatment, for the longest duration of available data.
5. Health services use: the proportion of participants who have hospital re-admission and/or primary care practitioner visits, for the longest duration of available data.
6. Adverse events: the proportion of participants who leave the study due to adverse events within 8 weeks of study commencement (range 4–16 weeks)

### Search strategy and study selection

As several systematic reviews have already been published in this area (e.g. [6–11]), we will conduct a hybrid overview of reviews [31] and systematic review methodology. We will find and extract RCTs and their associated data using the content of these reviews and the original RCT papers (including RCTs which are listed as not having being included in the final published reviews (e.g. [6, 9]). This will be supplemented by an updated search for RCTs, as is commonly done in other areas [41–43]. More precisely, we will search, from inception, the Cochrane Library (CENTRAL), CINAHL, MEDLINE/PubMed, EMBASE, MEDLINE In-Process, Epistemonikos and PsycInfo. Then, we will conduct a search of MEDLINE/PubMed and the Cochrane Library for recent RCTs published in the last 5 years (i.e. from 1st January 2014). We will also supplement this with a search of clinical trials registries: World Health Organization International Clinical Trials Registry Platform (WHO ICTRP) and clinicaltrials.gov, using similar terms. The reference lists for included RCTs will also be searched. Unpublished data will be requested for unpublished or ongoing studies, but data from abstracts only will be excluded. Searches will not be filtered by language, but non-English language articles will be omitted at the title screening stage. All search terms are provided in Additional file 2.

All references will be downloaded into EndNote or Reference Manager, and duplicates will be found and removed using the software automatic tools. Reviews and trials will be selected by FD and MD independently, with discussion as needed. If both reviewers agree that an RCT does not meet the inclusion criteria, it will be excluded from further analysis. Then, full texts of all eligible RCTs will be obtained and reviewed for final inclusion. Disagreements will be discussed with a third reviewer.

### Data extraction

RCT data will be extracted independently by FD and MD. We will adopt a structured data extraction form designed for the present review, to enhance the completeness and consistency of data extraction. Data extracted will include study characteristics (first author, year of publication, journal, setting), participant characteristics (sample size, mean age, % women, inclusion criteria [for CAD and depression], intervention details (anti-depressant [dose, duration, dosing schedule]; for psychotherapy interventions the TIDiER checklist headings will be adopted for extraction criteria (i.e. brief name of intervention, rationale/theory, materials, procedures, who provided the intervention, mode of delivery, location/setting, dose/intensity, tailoring, modifications, fidelity) [44]. While obviously the content of psychotherapies can be heterogeneous, the use of TIDiER headings will allow the careful documentation of any significant disparities in content and delivery forms that may necessitate subsequent sensitivity analysis, although it is difficult to estimate this a priori. TIDiER will also be used to fully describe the comparison groups, prior to grouping for the NMA analysis (see above). Outcomes data will also be extracted—see below for details. Summary effect sizes will be calculated from data extracted from the RCTs, including multi-arm (three or more groups) trials, where data will be extracted at the arm level from the original reports. Two review authors will verify that the data has been inputted correctly into the final dataset.

### Continuous outcomes

SMDs and 95% confidence intervals, or means, SDs and number of patients in each trial arm will be extracted for continuous outcomes. Otherwise, the authors will be emailed to request the data. If some data is omitted (e.g. missing SDs or if *p* values only are reported), we will use the *metaeff* command procedures in Stata to calculate SMDs and 95% confidence intervals from available data [45]. If insufficient data is available to calculate the SMD, we will include the study for descriptive purposes only and omit the study from the main network analyses. If insufficient data is available to calculate the 95% confidence intervals, we will impute this will the median from the other studies within that particular grouping. If > 5% of studies require such imputation, we will consider multiple imputation techniques. A sensitivity analysis will be conducted to determine if there are any important implications of such imputation.

### Binary outcomes

The numbers of participants in each arm with each event will be extracted for each trial arm. If data is unavailable, we will contact the authors. Values will be imputed where continuous data is provided instead of numeric values, using an approved method [46].

### Duration of RCTs and outcome assessments

For the synthesis of primary outcomes and the secondary outcomes of adverse events, we will adopt the 8-week threshold as per the previous review, or, if data is unavailable for this duration, we will use the closest available data from 4 to 16 weeks [13, 36]. If data on adverse events (dropout) is not available for this time period, we will consider using the overall dropout rate as a proxy. This was adopted as researchers believed that depression treatments should work in a clinically reasonable period of time. We will adopt a longer-term, 6-month depression assessment as a secondary outcome. For other outcomes, data from the longest duration of follow-up will be obtained.

### Missing RCT outcome data

We will primarily adopt the results as reported in the original trials, irrespective of whether they adopted appropriate methods for dealing with missing data or not (e.g. reporting per protocol results instead of intention-to-treat). Ratings of the appropriateness or otherwise of the missing data imputation is already part of the risk of bias assessment conducted in prior meta-analyses (e.g. [6, 11]).

### Unit of analysis

We will adopt the methods of the recent anti-depressant NMA when dealing with a unit of analysis issues [13, 36], if these were not addressed by the obtained meta-analyses. Data extracted for cluster RCTs will account for clustering in the design (e.g. we will use data from multi-level models), whereas data from only the first period of cross-over trials will be used, due to potential carryover effects.

### Risk of bias and quality ratings

Where available, we will extract the Cochrane risk of bias tool [47] ratings from prior reviews (e.g. [6, 11]). Where published reviews disagree on the quality rating, or where there is no such rating, FD and MD will independently assess included RCTs using this Cochrane risk of bias tool, with disagreements discussed and resolved with a third team member. Risk of bias will be assessed for the following design areas (for placebo-controlled trials): generation of allocation sequence, allocation concealment, blinding of outcome assessor, attrition (adopting similar criteria to Furukawa et al. [13, 36]), selective outcome reporting (for the primary outcomes only) and other domains (e.g. sponsorship bias). If any details are inadequate, RCT authors will be contacted for missing information. Given the nature of complex or psychotherapeutic interventions, assessing the blinding of treatment assignment is not usually possible, so it is likely that most of these trials will exhibit high risk of bias, whereas the placebo-controlled drug trials will be less biassed. We will also consider whether different risks of bias estimates are needed for particular arms of multi-arm trials [28]. When comparing the trials, the blinding of outcome assessments will take precedence [9].

We will also follow the recommendations for NMA and use the Grading of Recommendations Assessment, Development and Evaluation (GRADE) framework for obtained results [13, 36, 48]. This framework characterises the overall evidence contributing to the main outcomes from the network estimates incorporating information from the study limitations, imprecision, inconsistency, indirectness and publication bias, any of which could downgrade the obtained summary evidence [49]. GRADE ratings will be presented in a summary findings table.

### Statistical analysis

Descriptive statistics for the RCTs will be used to profile the overall study and clinical features, such as publication year, age, proportions of women, clinical setting, number of trial arms, intervention content and comparator group content. A network diagram will be used, with node size indicating the number of patients for each treatment (or comparator) group, and edge width used as an indicator of the numbers of studies making the comparison [20]. Several comparator groups will be used in the main network, as outlined in the ‘Comparison groups’ section above. The most influential network comparisons will be evaluated using a contribution matrix, which describes the proportion of the contribution to the entire network of each direct meta-analysis [50]. Two main networks will be evaluated using frequentist multivariate meta-analysis (commands *network meta* and *mvmeta*, which underpins the first command) in Stata 15 [51]. These commands perform restricted maximum likelihood methods for random effects multivariate meta-analysis by using a Newton-Raphson procedure, accounting for within- and between-study correlations. The assumptions of this model are that the multiple modelled effects represent a multivariate normal approximation of the estimated effects; that a multivariate linear regression can be performed due to linear effects between studies; a constant between-studies covariance matrix, where conditional variances of all components of the random effect are constant; and a symmetrical normal distribution which does not allow for light or heavy tails (which consequently means that outlier trial results can be overly influential for final estimates) [51]. The interested reader is referred to the following references for more detail [51, 52].

The first analysis will contain the original groupings as outlined above (i.e. pharmacotherapy, psychotherapies, exercise, collaborative care, comparator groups). A second main analysis will separate the different groupings, i.e. by type of anti-depressant (e.g. fluoxetine, sertraline), type of psychotherapy (e.g. CBT, interpersonal psychotherapy, mindfulness), if there is enough available data. Any derived ranking of treatments will only be done for primary outcomes, although given the probable sparsity of evidence we acknowledge in advance that this ranking may have a substantial error. As some trials may not report change scores, but may report end-of-trial scores only, we will consider a supplementary analysis where we include the end-of-trial only scores if this is required to generate the network, or there is likely to be substantial missing data (i.e. > 10% of trial estimates missing).

As interventions are by definition heterogeneous, random effects pairwise meta-analyses will be used to obtain SMDs or odds ratios (with associated 95% confidence intervals) for continuous and binary outcomes respectively [51, 53], with the *I*^2^ statistic used to quantify heterogeneity. All pairwise estimates and associated 95% confidence intervals will be reported. Pairwise meta-analytic estimates are usually reported in addition to the network estimates [13, 36, 52]. Among other reasons, they are useful (1) to determine the potential effects of any outliers and (2) to demonstrate any differences in estimated effects from the network meta-analysis which could be attributed to the correlation between the outcomes—which is ignored in a pairwise meta-analysis [52].

Transitivity is a key assumption of NMA and refers to the belief that an indirect comparison is a valid estimate of the unobserved direct comparison [48]. The transitivity assumption was initially addressed by the inclusion of patients with CAD only—as treatments for CAD are largely similar, i.e. risk factor control such as hypertension management, lipid-lowering and smoking cessation—these patients were considered similar enough for synthesising the information. Transitivity assessment will follow previous research [13, 36, 48]—by investigating effect modifiers across treatment comparisons, such as subthreshold depression or baseline depression severity, age, dosing (or intensity) [54–56], sex and cardiovascular disease severity [2], and differences in placebo-controlled versus other comparator group studies [39, 40]. Comparing the relative distribution of such variables across RCTs may provide some evidence for this assumption. Furthermore, we will compare the placebo-controlled trials to any head-to-head trials to ascertain any differences [36]. We assume that all RCT participants would have had equal opportunity to be randomised to any of the trial arms (apart from any potential patient preference studies). If transitivity is not demonstrated (e.g. if there are clear, statistically significant and/or clinically important differences in patients enrolled to trials in terms of age, sex, CAD or depression severity indices [48]), we may explore building separate networks to reflect the evidence.

Prior to conducting the NMA, inconsistency, which is the disagreement between direct and indirect evidence [48], will be assessed using both local and global methods in Stata as appropriate, and also by calculating the *I*^2^ for network heterogeneity and inconsistency [20, 28, 50, 57]. Heterogeneity variance will be considered equal within groupings, but possibly different among groups. Local parts of the network will be evaluated using the loop-specific and node-splitting methods. The global network will be evaluated using a design-by-treatment interaction model. We will also display inconsistency factors as recommended, but will use caution in interpretation due to the chance of finding inconsistency by chance alone, or have wide 95% confidence intervals [20].

Sensitivity analysis will also mainly follow previous work [13, 36]—the treatment effects for the primary outcomes will be explored in subgroup analysis and meta-regression for the following variables: study year, RCT sponsorship/funding (industry versus government/charity), baseline depression severity, intervention intensity or dosing schedule, comparator grouping, study design, setting or country. Where practicable, analyses will also be conducted addressing enrolment period: studies that enrol patients up to 6 months after an acute coronary event (or those that assess depressive symptoms on two or more occasions prior to enrolment) versus those that enrol stable patients or 6 months after an acute event; studies that provide depression treatment choice versus those that do not. Sensitivity analysis will address different levels of risk of bias (low, medium, high) [58], but also intensity of interventions as rated by a dichotomous variable (as rated by FD and MD, i.e. using the recommended to maximum doses of pharmacotherapies or greater than four sessions of psychotherapies—these will be classified as high-intensity interventions; otherwise, low dosage pharmacotherapy or four or fewer sessions of psychotherapy will be classed as low-intensity).

Funnel plots for NMA, which plot the difference between the study-specific effect sizes from the corresponding comparison-specific summary versus the inverted standard error, will be used to ascertain whether estimates from more imprecise RCTs are different from those RCTs with more precision (such as larger effect sizes for depression treatment in smaller studies) [28, 50]. A network meta-regression will investigate associations between effect size and study sample size.

Rankograms and surface under the cumulative ranking (SUCRA) curves will be used for treatment ranking [20]. SUCRA can be usefully re-expressed as the percentage of effectiveness/acceptability of depression interventions that would be rated first ranking, without uncertainty. Although we will report the probability that a given treatment is best, second best, third best etc., such probability statements will be interpreted cautiously unless there are actual clinically meaningful differences among the interventions.

All analyses will be implemented in Stata 15.

## Discussion

This will be the first NMA to provide a ranking of treatments for depression in those with CAD and should provide insights into the future development and implementation of the most promising effective methods. The adopted methodology, combining overview and comprehensive systematic review techniques for newer literature, as is common [41–43], should reduce duplication of effort yet provide a relatively quick and comprehensive answer to support clinical decision-making.

The results could show treating doctors whether it is better to initiate anti-depressant treatment, or refer for psychotherapy or other treatments, or both. Results should provide researchers with vital insights into effective interventions and funders with valuable information on the allocation of future research resources.

## Additional files

Additional file 1: PRISMA-P and NMA checklist items. (DOCX 28 kb)

Additional file 2: Search terms. (DOCX 22 kb)

## Abbreviations

CAD: Coronary artery disease
GRADE: Grading of Recommendations Assessment, Development and Evaluation
HRQoL: Health-related quality of life
NMA: Network meta-analysis
RCTs: Randomised controlled trials
SMD: Standardised mean difference
SUCRA: Surface under the cumulative ranking
TIDiER: Template for intervention description and replication checklist
WHO ICTRP: World Health Organization International Clinical Trials Registry Platform
