# Supplementary material for: Network meta-analysis of randomised trials of pharmacological, psychotherapeutic, exercise and collaborative care interventions for depressive symptoms in patients with coronary artery disease: hybrid systematic review of systematic reviews protocol
Source: Syst Rev. 2019 Mar 16;8:71. doi: 10.1186/s13643-019-0985-9 (PMC6420728; doi:10.1186/s13643-019-0985-9)
Supplement: Supplementary file 2 — Search terms. (DOCX 22 kb) [file 13643_2019_985_MOESM2_ESM.docx]

**Additional file 2: Proposed search terms for various databases**

Adapted from the following:

- Baumeister H, Hutter N, Bengel J. Psychological and pharmacological interventions for depression in patients with coronary artery disease. Cochrane Database of Systematic Reviews 2011, Issue 9. Art. No.: CD008012. DOI: 10.1002/14651858.CD008012.pub3
- MJ Best Practice filter for SRs, as - <https://bestpractice.bmj.com/info/toolkit/learn-ebm/study-design-search-filters/>

**CENTRAL, DARE, HTA and EED on The Cochrane Library**

#1 MeSH descriptor myocardial ischemia explode all trees

#2 MeSH descriptor Myocardial Revascularization explode all trees

#3 (ischemi* in All Text near/3 heart in All Text)

#4 (ischaemi* in All Text near/3 heart in All Text)

#5 (coronary in All Text near/3 disease* in All Text)

#6 angina in All Text

#7 myocardial next infarct* in All Text

#8 heart next infarct* in All Text

#9 (coronary in All Text near/3 bypass in All Text)

#10 (heart in All Text near/3 disease in All Text)

#11 (cardiac in All Text near/3 disease in All Text)

#12 chd in All Text

#13 cad in All Text

#14 (coronary in All Text near/3 angioplasty in All Text)

#15 (#1 or #2 or #3 or #4 or #5 or #6 or #7 or #8 or #9 or #10)

#16 (#11 or #12 or #13 or #14)

#17 (#15 or #16)

#18 MeSH descriptor depression explode all trees

#19 MeSH descriptor Depressive Disorder explode all trees

#20 MeSH descriptor Mood Disorders this term only

#21 “depression” in Keywords

#22 “depressive” in Keywords

#23 “Dysthymia” in Keywords

#24 dysthymi* in All Text

#25 (depressi* in All Text near/3 disorder* in All Text)

#26 (depressi* in All Text near/3 symptom* in All Text)

#27 mood next disorder* in All Text

#28 depression in Record Title

#29 antidepress* in All Text

#30 anti-depress* in All Text

#31 (#18 or #19 or #20 or #21 or #22 or #23 or #24 or #25 or #26 or #27)

#32 (#28 or #29 or #30)

#33 (#31 or #32)

#34 (#17 and #33)

**MEDLINE ALL (OVID)**

1 exp Myocardial Ischemia/

2 exp Myocardial Revascularization/

3 (isch?emi$ adj3 heart).tw.

4 (coronary adj3 disease).tw.

5 angina.tw.

6 myocardial infarct$.tw.

7 heart infarct$.tw.

8 (coronary adj3 bypass$).tw.

9 (heart adj3 disease).tw.

10 (cardiac adj3 disease).tw.

11 chd.tw.

12 CAD.tw.

13 (coronary adj3 angioplasty).tw.

14 or/1-13

15 Depression/

16 exp Depressive Disorder/

17 Mood Disorders/

18 dysthymi$.tw.

19 (depressi$ adj3 disorder$).tw.

20 (depressi$ adj3 symptom$).tw.

21 mood disorder$.tw.

22 affective disorder$.tw.

23 antidepress$.tw.

24 anti-depress$.tw.

25 or/15-24

26 14 and 25

27 randomized controlled trial.pt.

28 controlled clinical trial.pt.

29 randomized.ab.

30 placebo.ab.

31 drug therapy.fs.

32 randomly.ab.

33 trial.ab.

34 groups.ab.

35 or/27-34

36 (animals not humans).sh.

37 35 not 36

38 26 and 37

1. review.pt.
2. (medline or medlars or embase or pubmed or cochrane).tw,sh.
3. (scisearch or psychinfo or psycinfo).tw,sh.
4. (psychlit or psyclit).tw,sh.
5. cinahl.tw,sh.
6. ((hand adj2 search$) or (manual$ adj2 search$)).tw,sh.
7. (electronic database$ or bibliographic database$ or computeri?ed database$ or online database$).tw,sh.
8. (pooling or pooled or mantel haenszel).tw,sh.
9. (peto or dersimonian or der simonian or fixed effect).tw,sh.
10. (retraction of publication or retracted publication).pt.
11. or/39-48
12. 39 and 49
13. meta-analysis.pt.
14. meta-analysis.sh.
15. (meta-analys$ or meta analys$ or metaanalys$).tw,sh.
16. (systematic$ adj5 review$).tw,sh.
17. (systematic$ adj5 overview$).tw,sh.
18. (quantitativ$ adj5 review$).tw,sh.
19. (quantitativ$ adj5 overview$).tw,sh.
20. (quantitativ$ adj5 synthesis$).tw,sh.
21. (methodologic$ adj5 review$).tw,sh.
22. (methodologic$ adj5 overview$).tw,sh.
23. (integrative research review$ or research integration).tw.
24. or/51-61
25. 50 or 62
26. 63 and 38

**EMBASE (OVID)**

1 exp ischemic heart disease/

2 exp coronary artery surgery/

3 exp percutaneous coronary intervention/

4 (isch?emi$ adj3 heart).tw.

5 (coronary adj3 disease).tw.

6 angina.tw.

7 myocardial infarct$.tw.

8 heart infarct$.tw.

9 (coronary adj3 bypass$).tw.

10 (heart adj3 disease).tw.

11 (cardiac adj3 disease).tw.

12 chd.tw.

13 CAD.tw.

14 (coronary adj3 angioplasty).tw.

15 or/1-14

16 exp depression/

17 affective neurosis/

18 Mood Disorder/

19 dysthymi$.tw.

20 (depressi$ adj3 disorder$).tw.

21 (depressi$ adj3 symptom$).tw.

22 mood disorder$.tw.

23 affective disorder$.tw.

24 antidepress$.tw.

25 anti-depress$.tw.

26 or/16-25

27 15 and 26

28 controlled clinical trial/

29 random$.tw.

30 randomized controlled trial/

31 follow-up.tw.

32 double blind procedure/

33 placebo$.tw.

34 placebo/

35 factorial$.ti,ab.

36 (crossover$ or cross-over$).ti,ab.

37 (double$ adj blind$).ti,ab.

38 (singl$ adj blind$).ti,ab.

39 assign$.ti,ab.

40 allocat$.ti,ab.

41 volunteer$.ti,ab.

42 Crossover Procedure/

43 Single Blind Procedure/

44 or/28-43

45 (exp animals/ or nonhuman/) not human/

46 44 not 45

47 27 and 46

48 exp review/

49 (literature adj3 review$).ti,ab.

50 exp meta analysis/

51 exp Systematic Review/

52 or/48-51

53 (medline or medlars or embase or pubmed or cinahl or amed or psychlit or psyclit or psychinfo or psycinfo or scisearch or cochrane).ti,ab.

54 RETRACTED ARTICLE/

55 53 or 54

56 52 and 55

57 (systematic$ adj2 (review$ or overview)).ti,ab.

58 (meta?anal$ or meta anal$ or meta-anal$ or metaanal$ or metanal$).ti,ab.

59 56 or 57 or 58

60 47 and 59

**PsycINFO (1806 to present)**

As BMJ Best Practice does not provide filters for PsycInfo, we instead will adopt the recommended filter from University of Texas School of Public Health (search 40 below): <http://libguides.sph.uth.tmc.edu/search_filters/ovid_psycinfo_filters>

1 exp heart disorders/

2 heart surgery/

3 (isch?emi$ adj3 heart).tw.

4 (coronary adj3 disease).tw.

5 angina.tw.

6 myocardial infarct$.tw.

7 heart infarct$.tw.

8 (coronary adj3 bypass$).tw.

9 (heart adj3 disease).tw.

10 (cardiac adj3 disease).tw.

11 chd.tw.

12 CAD.tw.

13 (coronary adj3 angioplasty).tw.

14 or/1-13

15 exp affective disorders/

16 “DEPRESSION (EMOTION)”/

17 dysthymi$.tw.

18 (depressi$ adj3 disorder$).tw.

19 (depressi$ adj3 symptom$).tw.

20 mood disorder$.tw.

21 affective disorder$.tw.

22 antidepress$.tw.

23 anti-depress$.tw.

24 or/15-23

25 14 and 24

26 random$.tw.

27 ((singl$ or doubl$ or trebl$ or tripl$) adj25 (blind$ or dummy or mask$)).tw.

28 placebo$.tw.

29 crossover.tw.

30 assign$.tw.

31 allocat$.tw.

32 ((clin$ or control$ or compar$ or evaluat$ or prospectiv$) adj25 (trial$ or studi$ or study)).tw.

33 placebo/

34 treatment effectiveness evaluation/

35 mental health program evaluation/

36 experimental design/

37 clinical trials/

38 or/26-37

39 25 and 38

40 (((comprehensive* or integrative or systematic*) adj3 (bibliographic* or review* or literature)) or (meta-analy* or metaanaly* or "research synthesis" or ((information or data) adj3 synthesis) or (data adj2 extract*))).ti,ab,id. or ((review adj5 (rationale or evidence)).ti,ab,id. and "Literature Review".md.) or (cinahl or (cochrane adj3 trial*) or embase or medline or psyclit or pubmed or scopus or "sociological abstracts" or "web of science").ab. or ("systematic review" or "meta analysis").md.

41 40 and 39

**CINAHL Plus (EBSCO)**

As BMJ Best Practice does not provide filters for CINAHL, we instead will adopt the recommended filter from University of Texas School of Public Health (search 2 below): <http://libguides.sph.uth.tmc.edu/search_filters/cinahl_filters>

1

( (MH “Affective Disorders+”) or (TI depression) or dysthymi* or (mood disorder*) or (affective disorder*) or antidepress* or antidepress*

or (depressi* N3 disorder*) or (depressi* N3 symptom*) ) and ( (MH “Myocardial Ischemia+”) or (MH “Myocardial Revascularization+”)

or Angina or (myocardial infarct*) or (heart infarct*) or coronary or cardiac or chd or CAD or (heart disease) ) and ((MH “Clinical Trials+”) or randomi* or randomly or placebo* or trial )

2

(TI (systematic* n3 review*)) or (AB (systematic* n3 review*)) or (TI (systematic* n3 bibliographic*)) or (AB (systematic* n3 bibliographic*)) or (TI (systematic* n3 literature)) or (AB (systematic* n3 literature)) or (TI (comprehensive* n3 literature)) or (AB (comprehensive* n3 literature)) or (TI (comprehensive* n3 bibliographic*)) or (AB (comprehensive* n3 bibliographic*)) or (TI (integrative n3 review)) or (AB (integrative n3 review)) or (JN “Cochrane Database of Systematic Reviews”) or (TI (information n2 synthesis)) or (TI (data n2 synthesis)) or (AB (information n2 synthesis)) or (AB (data n2 synthesis)) or (TI (data n2 extract*)) or (AB (data n2 extract*)) or (TI (medline or pubmed or psyclit or cinahl or (psycinfo not “psycinfo database”) or “web of science” or scopus or embase)) or (AB (medline or pubmed or psyclit or cinahl or (psycinfo not “psycinfo database”) or “web of science” or scopus or embase)) or (MH “Systematic Review”) or (MH “Meta Analysis”) or (TI (meta-analy* or metaanaly*)) or (AB (meta-analy* or metaanaly*))

S1 AND S2

**WHO trials register -** [**http://apps.who.int/trialsearch/default.aspx**](http://apps.who.int/trialsearch/default.aspx)

Ischemi* AND depress* OR ischaem* AND depress* OR heart and depress* OR coronary AND depress* myocardi* AND depress* OR revascular* AND depress* OR angina AND depress* OR infarct* AND depress* OR bypass AND depress* OR cardiac AND depress* OR CHD AND depress* OR CAD AND depress*
